# Coming Soon: Disclosing the Identity of Donors by Genealogical Tests of Donor Offspring

**Published:** 2019

**Authors:** Mohammad Reza Sadeghi

Globally, more than 7 million live births now result from IVF cycles so that more than 6% of the European births and especially in Denmark about 10% of children are conceived through ART. Compliant with this increment, the infertility treatment using third-party and its success rate are increasing during the past decades. At present, more than 16% of the total IVF cycles, more than 20% of all live births result from oocytes, sperm, embryo donation and gestational carriers in US, so that the third-party IVF cycles are the main infertility treatment for aged couples and the success rate of ART cycles is 39.7% in live birth for third-party compared to 29.6% for autologous cycles (1).

The first sperm donation was performed in 1884 and the first donation of the oocyte was done almost one century later in 1983. Since that time, the most important aspects of third-party reproduction have always been the confidentiality in procedure and anonymity of donors. In that era, IVF clinics refused to disclose identity of donor and recommended to recipient couples not to disclose the usage of embryo or gamete donation to their children. Over the centuries, the same tendency and practice were common for adoption. Few parents left a child for adoption namelessly–so that the child and her/his adoptive couple had no data and background about abandoned child (2).

Anonymity is a multifaceted concept which is rarely continual and absolute. Anonymity expresses the rights and privacy of both donors and recipients. However, every child has a right to identify their biological parents and it should be regarded that these children never submit a consent form for their everlasting anonymity, and most of them will try to find their genetic parent in the future. But the rights, interests and privacy of other players of this scene (parents and donors) should not be ignored.

The widespread expansion of genetic databases is generally accompanied with the risk of identification of formerly anonymous donors. Voluntary DNA testing has been entirely common in recent years and so several huge genetic databases covering millions of genetic profiles are available worldwide. Although about 10 companies and websites collect biological samples and offer genealogy services, there is over 100 online DNA databases on medical DNA testing that most of them are available to community (3).

The ease of access to DNA ancestry and genealogical tests will change the concept of anonymity so that even the privacy of people who did not register may be threatened through searching in these accessible genetic databases. It is obvious that adoption facilities and IVF clinics active in donation should urgently change their guidelines, procedures, documents and counseling with donors and recipients to remove the word “anonymous” and substitute it with phrases and content that precisely describe the new situation for possible identification of anonymous donors and donor-conceived children in the future more easily and rapidly.

Accordingly, several countries such as Sweden as the pioneer and subsequently Austria, Finland, Iceland, Netherlands, Switzerland, UK, New Zealand and Australia removed donor anonymity. Following this change of approach, donation fee increased and the tendency to donation was slightly reduced and also the demography of donors has now shifted from young students to older men (2, 4).

This legislation allowed donor-conceived children to apply for identification of their donor through the court at legal age. Although the goal of anonymity is to protect the right of the donor, but donors should understand the possible short and long-term consequences of their donated gift. Although IVF clinics can still claim to keep secret the information of their donors from recipients and donor-conceived offspring, but the clinics have no authority on future facilities and technologies that allow us to search for genetics identity of individuals (4).

In this regard, the first DNA registry (UK donor link, UKDL) for all parties of donation (donor and donor offspring) was established in UK in 2004 for identification of each other through voluntary sharing of their complete data (5).

In the current situation, without DNA testing, they can be identified through DNA matching of their relatives who had DNA testing. At present, age, sex, and contact information of everybody are enough to find their DNA data in genetic databases.

This availability of DNA databases and genealogical tests enhances the risk to the accidental disclosure of donor or donor conceived children. Therefore, disclosure of biological heritage of donor conceived children is now considered a right in most countries. So many active groups support parents for disclosing the origin of donor conceived children at an early age to decrease the risk of accidental identification (2).

Finally, the duty of IVF clinics is very significant in counseling and advising infertile couples and donors about the future potential risks and right of conceived donor children in confronting with biological and legal parents. They should be correctly advised on privacy violation of all parties in the era of DNA ancestry and genealogical testing. Therefore, the donors who cannot accept the potential risk in the future should not be the donors whatsoever.
